# A study of the ultrasound-targeted microbubble destruction based triplex-forming oligodexinucleotide delivery system to inhibit tissue factor expression

**DOI:** 10.3892/mmr.2014.2822

**Published:** 2014-10-30

**Authors:** WEIHUA LIANG, WEIWEI ZHANG, SHIFU ZHAO, QIANNING LI, YIMING YANG, HUA LIANG, RONGCHUAN CENG

**Affiliations:** 1 Department of Neurology, Xinqiao Hospital, The Third Military Medical University, Chongqing 400038, P.R. China; 2 Deparment of Neurology, No. 263 Hospital of Beijing Military Region, Beijing 101149, P.R. China; 3 Deparment of Neurology, General Hospital of Beijing PLA Military Region, Beijing 100700, P.R. China; 4 Department of Internal Medicine, The Sixteenth Hospital of PLA, Altay, Xinjiang 836500, P.R. China; 5 Department of Internal Medicine, 66083 Clinic of Beijing Military Region, Beijing 102488, P.R. China

**Keywords:** tissue factor, oligonucleotides, ultrasonography, microbubble and gene transfer

## Abstract

The efficiency of cellular uptake of triplex-forming oligodexinucleotides (TFO), and the inhibition of tissue factor (TF) is low. The aim of the present study was to improve the absorption of TFO, and increase the inhibition of TF induced by shear stress both *in vitro* and *in vivo,* by using an ultrasound-targeted microbubble destruction (UTMD)-based delivery system. TFO-conjugated lipid ultrasonic microbubbles (TFO-M) were first constructed and characterised. The absorption of TFO was observed by a fluorescence-based method, and the inhibition of TF by immunofluorescence and quantitative polymerase chain reaction. ECV304 human umbilical vein endothelial cells were subjected to fluid shear stress for 6 h after treatment with TFO conjugated lipid ultrasonic microbubbles without sonication (TFO-M group); TFO alone; TFO conjugated lipid ultrasonic microbubbles, plus immediate sonication (TFO+U group and TFO-M+U group); or mock treated with 0.9% NaCl only (SSRE group). The *in vivo* experiments were established in a similar manner to the *in vitro* experiments, except that TFO or TFO-M was injected into rats through the tail vein. Six hours after the preparation of a carotid stenosis model, the rats were humanely sacrificed. The transfection efficiency of TFO in the TFO-M+U group was higher as compared with the TFO-M and TFO+U group (P<0.01). The protein and mRNA expression of TF in the TFO-M+U group was significantly decreased both *in vitro* and *in vivo* (P<0.01), as compared with the TFO-M, TFO+U and SSRE groups. The UTMD-based TFO delivery system promoted the absorption of TFO and the inhibition of TF, and was therefore considered to be favorable for preventing thrombosis induced by shear stress.

## Introduction

Triplex-forming oligonucleotides (TFOs) are a useful tool in anti-gene therapy due to their sequence-specific DNA binding capacity (1–4). The formation of triplexes with a targeted promoter inhibits the transcription of the target gene(5–7), resulting in control of gene expression. Although this strategy holds great potential, the rate of transcriptional inhibition remains a challenge.

Ultrasound-targeted microbubble destruction (UTMD) is a promising approach for effective thrombosis therapy. Previous studies (8–10) have confirmed that UTMD can enhance gene transfection efficiency. As a gene delivery system, UTMD can penetrate the endothelial barriers of the capillary walls, avoid initiating an immune response, and penetrate the nuclear membrane (11). Microbubble sonoporation has improved intracellular gene delivery (12–14) through the creation of transient pores in vascular endothelial cells, disruption of vascular endothelial integrity, and stimulation of endocytic cellular uptake (15). Co-administration of microbubbles and ultrasound, in combination with pharmaceutical thrombolysis *ex vivo,* can further enhance thrombolytic activity (16). Furthermore, the combination of microbubbles and ultrasound, without the use of fibrinolytics, increases the effect of ultrasound on thrombolysis *in vivo*. Administration of microbubbles has been shown to accelerate clot lysis during continuous 2-MHz ultrasound monitoring in stroke patients treated with intravenous tissue plasminogen activator (17). Previous studies (18–20) have further demonstrated that UTMD holds significant potential for thrombosis gene therapy.

Tissue factor (TF) (21–23) is a membrane-bound glycoprotein that is expressed or exposed at sites of vascular injury, and is essential to hemostasis. Binding of circulating factor VII/VIIa to TF initiates the clotting cascade, which promotes the formation of fibrin and platelet plugs. Activation of the TF gene endothelial domain (24–31) is usually induced in the narrow, curved, and divergent areas of brain blood vessels, and atherosclerotic plaques (32–34), suggesting that hemodynamic factors, including shear stress (SS), have an important role in cerebral atherosclerotic thrombosis and distribution (35–40).

In our previous studies, TFO (41–43) blocked the activation of the shear stress responsive element (SSRE) (44–48) in the TF gene promoter (44, 49–51) and resulted in the failure of TF gene transcription; however, the inhibition level of TF transcription by TFO still needs to be improved. The rate of TFO uptake in the ECV304 endothelial cell line, and the inhibition of TF in endothelial cells of the rat common carotid artery, 6.5 h after the injection of TFO, was 11.65 and 23%, respectively (41–43). The present study aimed to overcome the difficulty of delivering TFO into the cell nucleus by using a UTMD-based delivery system. TFO-conjugated lipid ultrasonic microbubbles were delivered for the first time using this method, both *in vitro* and *in vivo*.

## Materials and methods

### TFO design

The TFO sequence targeted to the SSRE in the promoter of the human TF gene *in vitro* was, 3′-GGGTGGTGTGGTGGGGGTGGG-5′. The TFO sequence targeted to the SSRE in the promoter of the rat TF gene *in vivo* was, 3′-GGGGGGTGGGGTGTGTGTGT-5′. The sequences were designed, synthesized and modified by a phosphorothioate method, and then labeled with fluorescein isothiocyanate (FITC). The synthesis, purification, modification, and fluorescence labeling was completed by Shanghai Shenggong Biological Engineering Technology & Services Co., Ltd. (Shanghai, China).

### Preparation of TFO conjugate lipid ultrasonic microbubble complexes

A suspension of lipid ultrasonic microbubbles, containing 7×10^9^ microbubbles/ml, was obtained from Xinqiao Hospital, The Third Military Medical University (Chongqing, China). TFO-FITC (10 μl, 100 μmol/l) and lipid ultrasonic microbubbles (100 μl, 7×10^9^ μg/ml) were gently agitated in phosphate-buffered saline (PBS), and the resulting transfection complexes were transferred to a polystyrene tube and incubated at 4°C overnight. The shape of the complexes was observed using a light microscope, and the fluorescence labeling was detected using a fluorescence microscope. The particle size, diameter, and surface potential was detected using a Coulter events-per-unit-time meter and a Malvern laser particle size analyzer (Zetasizer 3000; Malvern, Westborough, MA, USA).

### Antibodies and cell culture

A polyclonal rabbit anti-rat TF primary antibody and rhodamine labeled anti-rabbit secondary antibody were obtained from Boster Biological Technology Co., Ltd. (Wuhan, China.)

ECV304 human umbilical vein endothelial cells were obtained from the China Center for Type Culture Collection (Wuhan, China) and incubated in M199 medium with 10% fetal bovine serum at 37°C in a humidified environment of 5% CO_2_ and 95% air. The initial cell viability was determined for further experiments.

### In vitro experimental protocol

The TFO-conjugated lipid ultrasonic microbubbles were centrifuged at low speed prior to the experiment. The suspension was diluted with 0.9% NaCl to a final concentration of 0.2 μmol/l and a final volume of 60 μl. The ECV304 cells were randomly divided into four groups; a blank control group (SSRE group), in which the ECV304 cells were mock treated with 0.9% NaCl without TFO, microbubble or ultrasound; a TFO and ultrasound group (TFO+U), in which the ECV304 cells were added to the TFO mixture and immediately sonicated using a therapeutic ultrasound transducer (Xinqiao Hospital, The Third Military Medical University) with parameters set at 1 MHz, 1 W/cm^2^, 30 S, and a duty cycle of 0.5%; a TFO conjugated-lipid ultrasonic microbubble group (TFO-M), in which the ECV304 cells were added to the TFO conjugated lipid ultrasonic microbubbles; and a TFO-conjugated lipid ultrasonic microbubble injection plus ultrasound (U) group (TFO-M+U), in which the ECV304 cells were added to the TFO conjugated lipid ultrasonic microbubbles and exposed to ultrasound with the same irradiation parameters as the TFO+U group. The ECV304 cells were then subjected to fluid shear stress of 12 dyn/cm for 6 h.

The efficacy of the gene transfection was measured as the number of fluorescent cells per region, and then normalized to the total number of cells per unit area. All of the fluorescent cells within the region of insonation were counted, as well as the total number of cells present by both phase contrast and fluorescence microscopy. Six OptiCells^®^ (China Center for Type Culture Collection, Wuhan, China) were used per treatment group and the experiment was repeated at least twice on separate days.

The inhibition of TF gene expression was measured 6 h after application of fluid shear stress of 12 dyn/cm. The expression of TF protein was detected using an immunofluorescence method. The samples were washed with PBS and fixed with cold 4% paraformaldehyde. The samples were then incubated with polyclonal rabbit anti-rat TF primary antibodies (1:400) at 4°C overnight, followed by incubation with rhodamine-labeled anti-rabbit secondary antibodies (1:200) at 37°C for 1 h; all antibodies were obtained from Boster Biological Technology Co. Ltd (Wuhan, China). A laser scanning confocal microscope was then used to examine the expression and distribution of fluorescence in the ECV304 cells. Image Pro Plus (MediaCybernetics, Inc., Rockville, MD, USA) analysis system was used to determine the average gray scale of positive expression. The expression of TF mRNA was analyzed by quantitative polymerase chain reaction (qPCR). Total RNA was isolated from the cultured ECV304 cells using TRIzol (Invitrogen Life Technologies, Carlsbad, CA, USA) reagent according to the manufacturer’s instructions. Primer sequences for TF were; forward, 5′-GAACCCAAACCCGTCAAT-3′ and reverse, 5′-GAAGACCCGTGCCAAGTA-3′. The reverse transcription was performed at 42°C for 40 min and the cDNA (2 μl) was amplified under standard PCR reaction conditions. The qPCR reaction was performed as follows: 5 min at 94°C (one cycle), 30 sec at 94°C, 30 sec at 55°C, 30 sec at 72°C, plate reading (38 cycles), and then 10 min at 72°C. The PCR amplification was performed on a thermal cycler over 27 cycles. The average gray value was analyzed using Gel Pro Analyzer (MediaCybernetics) software and a raw data value for TF expression in each group was normalized to GAPDH.

### Establishment of a Sprague Dawley (SD) rat model of carotid artery stenosis

SD rats were housed in a constant room temperature of 24°C under a 12 h light-dark cycle, and fed *ad libitum*. All experiments were performed with the approval of the Third Military Medical University Animal Ethics Committee. Efforts were made to minimize animal suffering and to keep the number of animals used to a minimum. Twenty-four male SD rats aged 6 months old and weighing 300±8.5 g were randomly placed into one of the four groups (n=6). The rats were anaesthetized by intraperitoneal injection of 3% pentobarbital sodium at a dose of 45 mg/kg until the eyelash reflex disappeared. The rats were then fixed in a dorsal position. Using aseptic techniques, a 2cm incision was made in the median neck of each rat. Following layer separation, ~1 cm of the left common carotid artery was separated from the paratracheal carotid sheath, set into a longitudinally-split silica gel tube with an inner diameter of 0.5 mm and a length of 3 mm, tightly ligated twice using no. 4 silk thread, and sewn onto the skin following repositioning.

### In vivo experimental protocol

The TFO-conjugated lipid ultrasonic microbubble was centrifuged at low speed prior to the experiment and the suspension was diluted with 0.9% NaCl to a concentration of 1.0 mg ml^−1^. The rats were anesthetized using 3% sodium pentobarbital and were fixed on the experimental table. All the TFOs or mock complexes (0.5 mg kg^−1^) were administered through the tail vein. The carotid stenosis animal model was generated 0.5 h after treatment.

The SD rats were intravenously injected with or without ultrasound (U) treatment to derive the following four groups: 0.9% NaCl only without TFO, microbubble, or ultrasound (blank control SSRE group); half mg kg^−1^ of TFO plus immediate sonication with a therapeutic ultrasound transducer set at 347 KHz and 2.4 MPa for 2 min (TFO+U group); TFO conjugated lipid ultrasonic microbubbles (TFO-M group); and TFO-conjugated lipid ultrasonic microbubble plus ultrasound with the same sonication parameters as above (TFO-M+U group). Half an hour after the treatment, the carotid stenosis model was generated and six hours after model preparation, the rats were humanely sacrificed. Serial sections of the left common carotid artery were perfused with 0.9% NaCl solution at a velocity of 3 ml/min under low pressure until the outflow of liquid was transparent. The liquid was then changed to 100 ml paraformaldehyde (4%), diethylpyrocarbonate (0.1%), and PBS (0.1 M) under low pressure to perform *in situ* perfusion and fixation. Subsequently, the stenosis segment was dissected from the left common carotid artery and embedded in embedding medium. Frozen 5-μm sections were then subjected to immunofluorescence. The samples were incubated with polyclonal rabbit anti-rat TF primary antibody and rhodamine labeled anti-rabbit secondary antibody. A laser scanning confocal microscope was used to examine the expression and distribution of fluorescence in the frozen sections and the Image Pro Plus (MediaCybernetics) was used to determine the average gray scale of expression.

### Statistical analysis

Statistical analyses were performed using SPSS version 13.0 (SPSS, Inc., Chicago, IL, USA). All values are expressed as means ± standard deviation. Analysis of variance was used to determine significant differences through multiple comparisons. A P<0.05 was considered to indicate a statistically significant difference.

## Results

### Preparation of TFO conjugated lipid ultrasonic microbubble complexes

**Figure 1:**
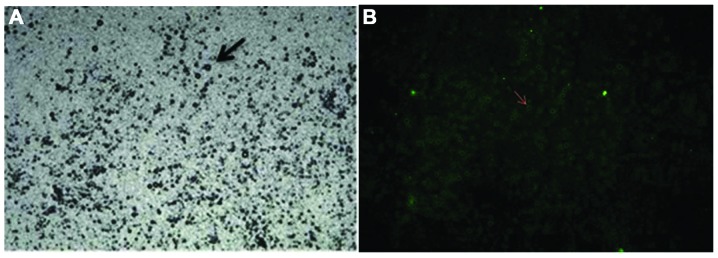
Visualisation of FITC-labeled TFO lipid microbubbles. (A) Lipid microbubbles labeled with FITC-TFO were observed under a light microscope (magnification, ×100). The arrow indicates one of the lipid ultrasonic microbubbles labeled with FITC-TFO. (B) Lipid microbubbles labeled with FITC-TFO under a fluorescence microscope (magnification, ×100). The red arrow indicates one of the lipid ultrasonic microbubbles labeled with FITC-TFO. FITC, fluorescein isothiocyanate; TFO, triplex-forming oligonucleotides.

**Figure 2:**
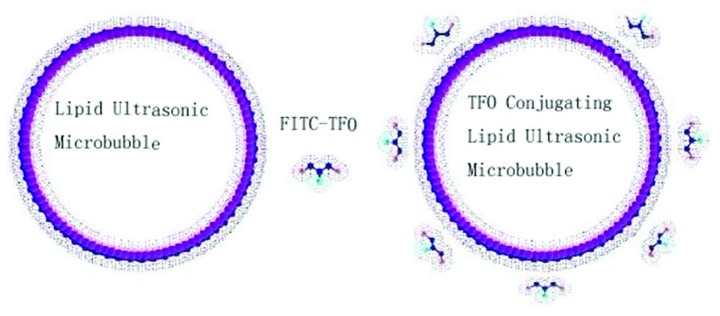
Model of TFO conjugated lipid ultrasonic microbubbles. TFO, triplex-forming oligonucleotides.

The lipid ultrasonic microbubble with FITC-labeled TFO appeared as a pale green suspension, and had a smooth round surface, even size and light density as observed under a light microscope (Fig. 1A). The microbubble concentration was ~7×10^9^/ml. The microbubble surfaces appeared green under the fluorescence microscope (Fig. 1B), while the lipid microbubbles without FITC-TFO were not visible, indicating that FITC-TFO was packaged on the microbubble lipid membrane (Fig. 2). The analysis of the particle size and diameter indicated that the mean intensity, volume and mean diameter were 2092.8, 2114.2, and 2166.9 nm, respectively. The surface potential analysis was −46.0±1.6 mm.

### UTMD-based TFO delivery system in vitro

**Figure 3:**
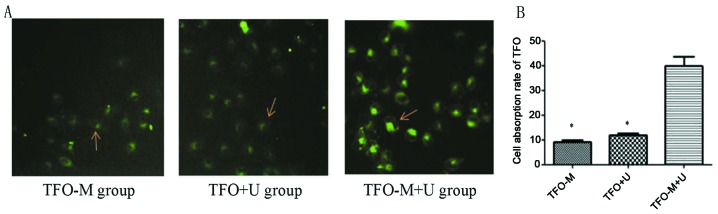
Cell absorption rate of TFO. (A) The rate of TFO absorption by ECV304 cells was detected by fluorescence microscopy (magnification, ×400). The red arrows indicate positive green fluorescence of FITC-labeled TFO in ECV304 cells. (B) The rate of TFO absorption by ECV304 cells in the TFO-M, TFO+U and TFO-M+U groups. The values represent the means ± standard deviation, n=6 per group. *P<0.01 as compared with the TFO-M+U group. TFO, triplex-forming oligonucleotides; M, microbubble; U, ultrasound.

The absorption rate of TFO into ECV304 cells was measured by fluorescence microscopy. The green fluorescence of FITC-labeled TFO was detected in the ECV304 cells (Fig. 3A), and was visible in the three experimental groups. The positive cells were most abundant in the TFO-M+U group as compared with the TFO-M and TFO+U group. The green fluorescence signal in the TFO-M and TFO+U groups was weak and mainly distributed in the cytoplasm, whereas the TFO-M+U group exhibited brighter green fluorescence. The transfection efficiency of TFO in the TFO-M+U group (38.83±6.52%) was significantly higher as compared with the TFO-M (9.50±2.88%) and the TFO+U group (12.66±3.01%, P<0.01) (Fig. 3B). There was no significant difference between the TFO-M and TFO+U groups (P>0.05).

**Figure 4:**
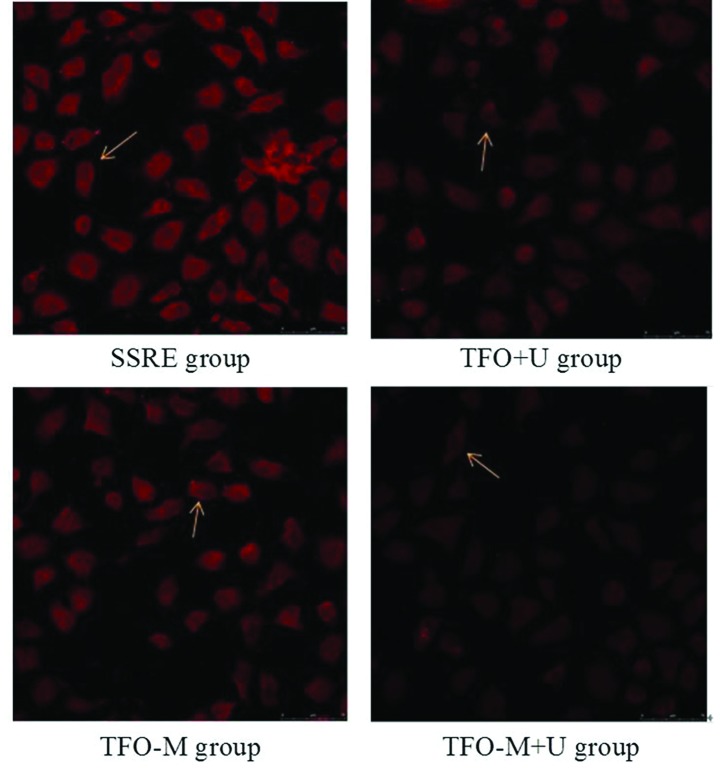
Immunofluorescent analysis of tissue factor protein *in vitro* (magnification, ×400). The arrows indicate positive immunofluorescent detection of tissue factor protein. TFO, triplex-forming oligonucleotides; M, microbubble; U, ultrasound; SSRE, shear stress response element.

The expression of TF protein was detected by immunofluorescence as fine red particles (Fig. 4). The TF protein was observed mainly in the cytoplasm and membrane of the ECV304 cells in the SSRE group, with a small amount in the nucleus. The intensity of the red fluorescence was greater in the SSRE group as compared with the TFO+U, TFO-M and TFO-M+U groups; the intensity in the TFO-M+U group was significantly lower as compared with the TFO+U and TFO-M groups. The TF protein content in the TFO+U (36.83±8.34), the TFO-M (40.77±9.40) and the TFO-M+U groups (13.98±6.39) was significantly lower as compared with the SSRE group (74.00±16.67) (P<0.01). The gray value in the TFO-M+U group was significantly lower as compared with both the TFO+U or TFO-M group (P<0.01); and there was no significant difference between the TFO+U and TFO-M groups (P>0.05).

**Figure 5:**
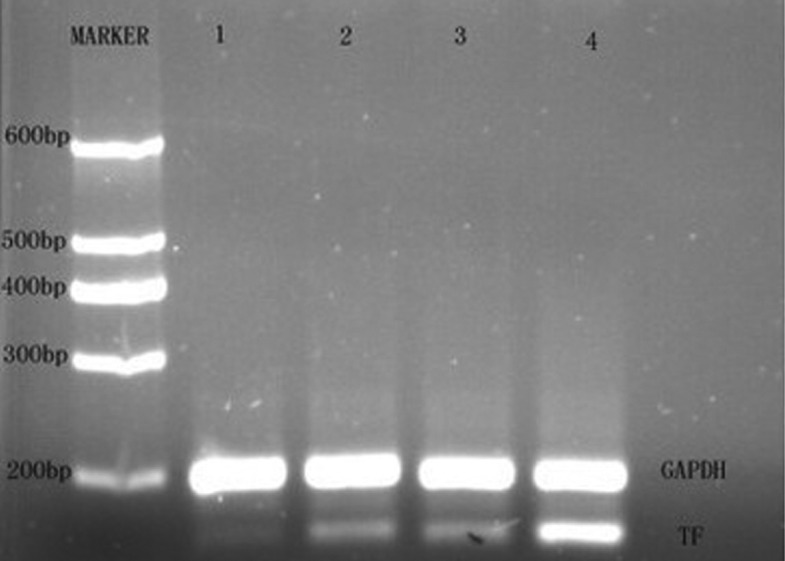
Analysis of tissue factor mRNA expression by quantitative polymerase chain reaction. Lane 1, TFO-M-U group; Lane 2, TFO-M group; Lane 3, TFO-U group; Lane 4, SSRE group. TFO, triplex-forming oligonucleotides; M, microbubble, U, ultrasound; SSRE, shear stress response element; TF, tissue factor; bp, base pairs.

TF mRNA expression was determined by qPCR (Fig. 5). There was a marked amplification of TF in the SSRE group. Based on image analysis, the TF mRNA was significantly lower (P<0.01) in the TFO+U (0.36±0.07), the TFO-M (0.38±0.07) and the TFO-M+U groups (0.11±0.02), as compared with the SSRE group (0.71±0.08). The TF mRNA expression in the TFO-M+U group was significantly lower as compared with the TFO+U and TFO-M group (P<0.01), however there was no significant difference between the TFO+U and TFO-M group.

### UTMD-based TFO delivery system in vivo

**Figure 6:**
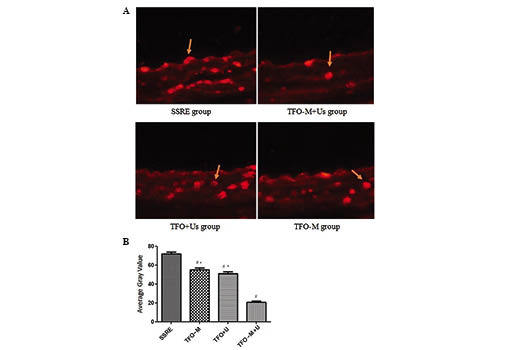
Immunofluorescence assay of tissue factor protein *in vivo*. (A) *In vivo* expression of tissue factor protein was detected by immunofluorescence microscopy (magnification, ×400). The arrows indicate positive immunofluorescence of tissue factor protein in endothelial cells of the carotid artery. (B) Average gray value in the SSRE, TFO-M, TFO+U and TFO-M+U groups. The values represent the means ± standard deviation, n=6 per group. ^#^P<0.01 as compared with the SSRE group; ^*^P<0.01 as compared with the TFO-M+U group. TFO, triplex-forming oligonucleotides; M, microbubble; U, ultrasound; SSRE, shear stress response element.

A rat model of carotid stenosis was successfully generated. The expression of TF protein in endothelial cells of carotid arteries in the four different groups was detected by immunofluorescence (Fig. 6A). The number of positive cells and the degree of staining was significant in the SSRE group, as compared with the other groups. The amount of red fluorescence in the TFO+U, TFO-M and the TFO-M+U group was lower; with the amount of fluorescence in the TFO-M+U group being significantly lower as compared with the TFO+U and TFO-M groups. Image analysis identified that the TF protein content in the TFO+U (51.22±5.69), TFO-M (55.22±6.47) and the TFO-M+U groups (20.59±4.38) was significantly lower (P<0.01) as compared with the SSRE group (71.78±7.10) (Fig. 6B). The fluorescence in the TFO-M+U group was significantly lower as compared with the TFO+U and TFO-M groups (P<0.01) and there was no significant difference between the TFO+U and TFO-M groups.

## Discussion

To improve the efficiency of TFO delivery, a UTMD-based delivery system was used to deliver TFO both *in vitro* and *in vivo*. It was first observed that FITC-labeled TFO had been successfully packaged onto the lipid microbubble membrane, and surface potential analysis showed that the FITC-labeled TFO microbubble measured −46.0±1.6 mm. The average size of the microbubble contrast agent has a key role in function. The microbubbles must be small enough to pass through the capillary wall endothelial barriers and be less than the diameter of a human red blood cell (7.8 μm) (52). The typical diameter range of the microbubbles was 0.5–10 μm, with some microbubbles reaching nanoscale.

The absorption of TFO and the inhibition of TF by the UTMD-based delivery system was observed *in vitro*. These results indicated that UTMD efficiently delivered TFO to the cells, resulting in a down regulation of TF protein and mRNA expression. The decrease in TF expression was associated with increased TFO in ECV304 cells *in vitro*. The increased TFO transfection efficiency was associated with a decrease in TF expression, suggesting that TFO was effective in inhibiting TF expression induced by shear stress in ECV304 cells. The UTMD-based TFO gene delivery system could significantly increase the absorption rate of TFO into cells and subsequently strengthen the inhibition of TF expression *in vitro*.

The expression of TF induced by shear stress in endothelial cells of rat carotid arteries, was observed following UTMD-based TFO delivery *in vivo*. The results from the present study were in accordance with previous studies: TFO formed triplexes with the TF endothelial cell gene promoter region *in vitro* (53) and *in vivo* (26). TFO uptake and TF inhibition in the ECV304 endothelial cell line and of the rat common carotid artery, 6.5 h after the injection of TFO, were ~11.65% and 23% TF transcription, respectively (41–43). TFO absorption in the TFO-M+U group was increased to 38.83% *in vitro* and the inhibition level of TF in the TFO-M+U group was decreased to 71.31%. These findings suggest that TF expression, induced by fluid shear stress of the cells of the carotid artery, could be inhibited by TFO. The UTMD-based TFO gene delivery system could promote inhibitive effects, and may be favorable for preventing shear stress-induced thrombosis *in vivo*.

Taken together, the UTMD-based TFO delivery system increased TFO delivery and decreased TF expression *in vitro* and *in vivo*. However, the amount of TFO loaded on the lipid ultrasonic microbubbles is limited, and the optimization of ultrasound parameters and strategies, in order to increase TFO absorption, may be time-consuming. The lipid ultrasonic microbubble may be used as a carrier for TFO, and the UTMD-based TFO delivery system provides a promising strategy for cerebral thrombosis gene therapy.
